# Improving safety and communication for healthcare providers caring for SARS-COV-2 patients

**DOI:** 10.1186/s12245-022-00464-y

**Published:** 2022-11-12

**Authors:** Ahmed Taher, Peter Glazer, Chris Culligan, Stephanie Crump, Steven Guirguis, Jennifer Jones, Alia Dharamsi, Lucas B. Chartier

**Affiliations:** 1 University Health Network, 200 Elizabeth Street R. Fraser Elliott Building, Ground Floor, Room 480, Toronto, ON M5G 2C4 Canada; 2 Department of Medicine, Division of Emergency Medicine, University of Toronto, C. David Naylor Building, 6 Queen’s Park Crescent West, Third Floor, Toronto, ON M5S 3H2 Canada; 3 Department of Family and Community Medicine, Division of Emergency Medicine, University of Toronto, 500 University Avenue, Fifth Floor, Toronto, ON M5G 1V7 Canada

**Keywords:** Patient safety, Healthcare providers, Safety, SARS-CoV

## Abstract

**Background:**

Decreasing healthcare provider (HCP) exposure to the severe acute respiratory syndrome coronavirus 2 (SARS-COV-2) virus in emergency departments (EDs) is crucial. Approaches include limiting the HCP presence and ensuring sealed isolation rooms, which can result in communication difficulties. This quality improvement (QI) initiative aimed to decrease by 50% duration of isolation room door opening and increasing HCP-perceived communication clarity by one point on a five-point Likert scale.

**Methods:**

This was a prospective, multi-stage project with three Plan-Do-Study-Act (PDSA) cycles between May and July 2020: (1) an educational intervention, (2) the introduction of a novel transceiver communication device, and (3) utilizing a clinical champion. Statistical Process Control XbarR charts were used to assess for special cause variation, and two-tailed Mann-Whitney *U* tests were used for statistical significance between Likert survey means. Qualitative responses underwent thematic analysis.

**Results:**

Observation of 174 patient encounters was completed over 33 days, with 95 meeting the inclusion criteria. Door opening decreased from baseline (*n*=40; mean 72.97%) to PDSA 3 (*n*=21; mean 1.58%; *p*<0.0001). HCP-perceived communication clarity improved from baseline (*n*=36; mean 3.36) to PDSA-3 (*n*=49; mean 4.21; *p*<0.001). Survey themes included positive effects on communication and workflow, with some challenges on the integration of the new device into the clinical workflow. HCP-perceived errors, workarounds, and workflow pauses showed significant improvements.

**Conclusion:**

This QI initiative with a novel transceiver showed significant decreases in isolation room door opening and increases in communication clarity. Future work will expand to operating rooms and intensive care units.

**Supplementary Information:**

The online version contains supplementary material available at 10.1186/s12245-022-00464-y.

## Background

The severe acute respiratory syndrome coronavirus 2 (SARS-COV-2) pandemic has caused over 173 million cases worldwide, with over 3.7 million deaths, by June 2021 [1]. Healthcare provider (HCP) infections are thought to comprise a notable proportion of these cases [2]. The emergency department (ED) is a high-risk setting of exposure to SARS-COV-2, especially given the performance of aerosol-generating medical procedures (AGMPs) such as endotracheal intubation [3]. Therefore, EDs have sought to follow international guidance on personal protective equipment (PPE) practices [4], increase isolation rooms [5], utilize dedicated intubation teams [6] [7], increase the use of ED telemedicine [8], and implement updated resuscitation guidance [9].

Patients with SARS-COV-2 requiring intubation [10] represent a risk to HCPs [11] given the potential aerosolization of the virus [12]. The risk is increased, in part because of the duration of exposure and proximity to the patient increase [13]. Therefore, it is important to minimize the door opening of AGMP patients, which may transmit aerosolized material and to minimize the number of HCPs present inside isolation rooms. It is also necessary to minimize doffing and re-donning of PPE, which produces self-contamination risks and over-use of resources [14].

Given the importance of maintaining closed isolation room doors, it is important to continue clear communication between HCPs across closed doors. This presented multiple challenges for our ED during the first wave of the pandemic, which prompted multiple in situ simulation exercises [15] to prepare HCPs and increase safety practices with AGMPs. A variety of communication methods across the closed doors were trialed, including a “call bell” system (paging the nurse desk), a whiteboard and marker through glass windows, and finally a commercial baby monitor system, which was in use during our baseline period. Communication remained the most common challenge cited in debriefs after ED resuscitations.

Given the communication challenges across isolation rooms along with the risks of the continued door opening and potential doffing and re-donning, we conducted a quality improvement (QI) initiative with the aim to decrease isolation room door opening and improve HCP communication clarity.

## Methods

### Study setting

Toronto General Hospital (TGH) is a quaternary care adult academic 371-bed medical center part of the University Health Network in Toronto, Ontario, Canada. TGH is a referral center for SARS-COV-2 critically ill patients. The ED sees approximately 55,000 patients per year. The TGH ED has 24 beds in the acute zone, of which 17 are isolation rooms. Isolation rooms have glass doors with curtains outside them (for privacy) or anterooms leading to the patient room. Baseline communication with the outside team was through door opening or by using a commercial baby monitor system.

### Participants

Our HCP participants consisted of ED nurses, physicians, and trainees on shift during the data collection period. Patient encounters were included if the patient was greater than 16 years old, placed in an isolation room, and met any of the potential infectious symptoms for SARS-COV-2 (Additional file 1: Appendix 1), regardless of presentation acuity.

### Study design

This QI initiative was completed in a prospective, multi-stage approach consisting of a baseline period and three discrete Plan-Do-Study-Act (PDSA) cycles. The SQUIRE 2.0 Guidelines were used for study design and reporting [16]. We received a formal exemption from our organization’s research ethics board. We were supported by a local grant: The Mount Sinai Hospital - University Health Network (MSH-UHN) Academic Medicine Organization COVID-19 Innovation Grant.

### Interventions

An initial period of stakeholder engagement was undertaken at daily nursing huddles and ED physician business meetings, which helped devise three sequential PDSA cycles. PDSA-1 was an educational intervention, whereby HCPs were educated about the need and rationale for closing isolation room doors through email and daily departmental huddles.

**Fig. 1:**
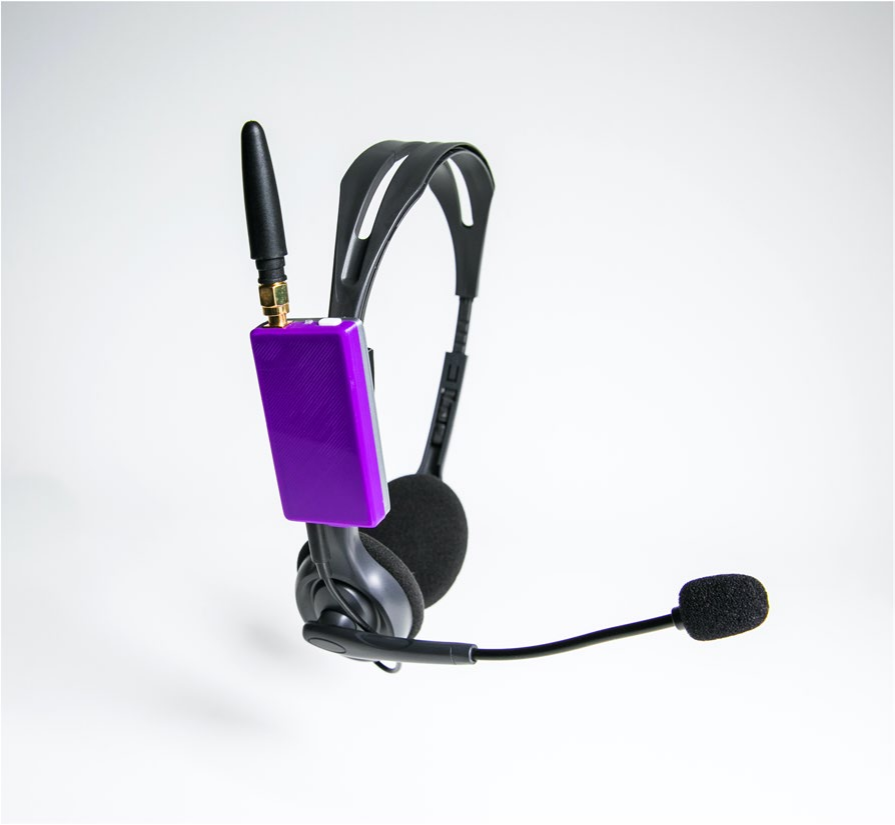
Transceiver headset

Given the limitations of educational interventions, concurrent search was done for a better information communication technology (ICT). PDSA-2 was the introduction of an ICT developed by a co-author (CC) called the *TQC 200* “the transceiver” [17]. The transceiver was initially developed for use in sports but was adapted to our local ED setting to replace the existing use of the commercial baby monitor system. The transceivers are wireless radiofrequency wearable paired headsets that allow for two-way uninterrupted communication across closed isolation room doors (Fig. 1). HCPs entering an isolation room would wear one transceiver headset as part of the PPE donning process, and the remaining team members would wear paired transceiver headsets. Team members would then be able to talk to each other freely without pushing any buttons. Devices would be disinfected according to approved infection prevention and control protocols and plugged in to recharge in between use. PDSA-3 was mainly to embed and sustain the change that was noted. It included a local clinical champion (registered nurse) who was identified to demonstrate, remind, and support nurses and physicians in their use of the new technology available. During this phase, further refinement of the transceiver was also made based on ongoing clinician feedback.

### Data collection

A dedicated research coordinator (SG; not involved in transceiver development) directly observed patient encounters that met the inclusion criteria throughout the baseline period and PDSA cycles. The total number of minutes HCPs spent during a clinical encounter (an episode of going into the room until exiting it) and the total number of minutes of door opening during that encounter were recorded. Data collection consisted of a convenience sample that occurred during research coordinator availability, between 09:00 and 15:00 on weekdays, between May and July 2020.

Baseline surveys were distributed to nursing staff and included quantitative (Likert scale) and qualitative (two open-ended questions) portions. Following the conclusion of PDSA 3, a follow-up survey was collected (Additional file 1: Appendix 2). Likert scale surveys included rating communication clarity, communication errors, the need for opening isolation doors to communicate, using other means to communicate (e.g., hand gestures, whiteboard), or having to alter clinical workflow to communicate adequately. The follow-up survey included questions comparing the baby monitor system to the transceiver. Finally, open-ended questions were asked about the team’s communication approach. Surveys were collected in the ED during HCP shifts. All surveys were developed locally and piloted with a sample of HCP prior to administration. Transceiver-specific qualitative data was collected during PDSAs 2 and 3 to allow for rapid improvements during the corresponding PDSA cycle.

### Outcome and balancing measures

The primary outcome measure was the duration of the isolation room door opening, which was the percentage of time the isolation room door was left open between the HCP(s) entering and exiting the room for that corresponding patient encounter. Our aim was to decrease isolation room door opening by 50% over a period of 3 months. Our secondary outcome measure was HCP-perceived communication clarity across closed isolation room doors. This was measured using a Likert scale survey. Our aim was to increase HCP-perceived communication clarity using an ICT by one point on a five-point Likert scale over a period of 3 months.

Our balancing measures were HCP-perceived communication errors across the closed isolation room door during patient encounters, perceived use of workarounds across the closed door (e.g., hand gestures), and HCP pausing their workflow to communicate with team members outside rooms (all using a Likert scale). We also measured the percentage of patients seen in the ED who were placed in isolation rooms, i.e., failed triage screen presenting with one or more infectious symptoms to monitor the infectious landscape.

### Data analysis

We utilized Statistical Process Control ([SPC] or Shewhart) XbarR charts [18] to assess for special cause variation. SPC charts were completed with QI Macros© (Version 2018.04, KnowWare International Inc., Denver, CO, USA) for Microsoft© Excel© (Microsoft Corporation, Redmond, WA, USA, Version 14.5.9). Centerline calculation utilized formulae [19] and control limits utilized rules recommended by the Institute for Healthcare Improvement [20]. Four discrete periods of data were collected (baseline and three PDSA cycles). For the baseline and follow-up surveys, we used a two-tailed Mann-Whitney *U* test to assess for statistical significance between means, with a significance level set at *p*<0.05. The qualitative (open-ended questions) underwent thematic analysis [21].

Exclusion criteria for points used to create the SPC charts included: subgroups with less than three data points according to accepted rules [22], patients who passed the triage infectious assessment (no infectious symptoms reported), patient interactions lasting less than 5 min (chosen as a minimum to include meaningful clinical interaction), and patient interactions when HCP were discharging patients from the ED (e.g., disconnecting from monitors and patient leaving).

## Results

**Fig. 2:**
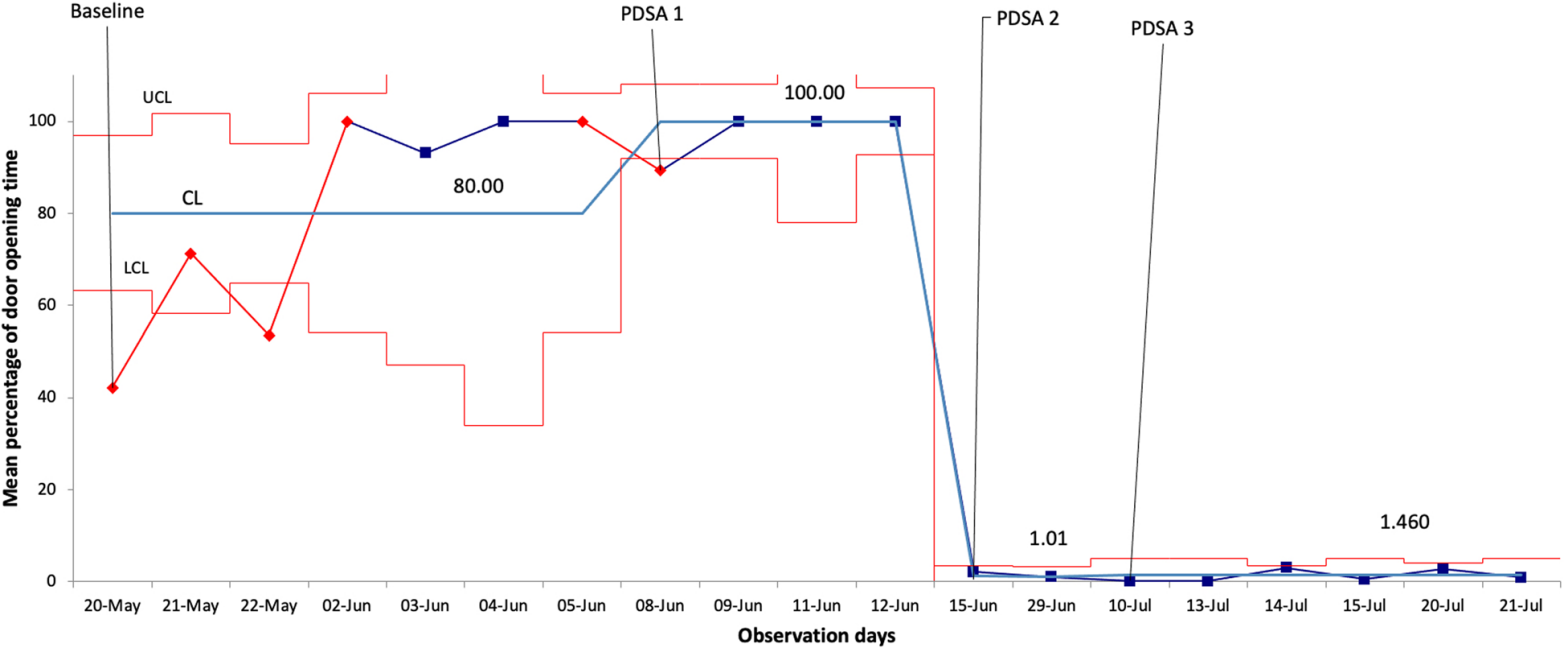
Isolation door opening XbarR statistical process control chart. CL center line; UCL upper control limit; LCL lower control limit; PDSA Plan-Do-Study-Act; medians are adjacent to the control limits

Direct observation of 174 patient encounters was completed over 33 days. Ninety-five encounters were included in the final analysis, while 79 met exclusion criteria and were removed from the analysis (38 did not fail the infectious screen; 25 encounters were less than 5 min; 16 subgroup days had less than three data points). Door opening (primary outcome) increased from baseline (*n*=40) with a mean of 72.97 to 96.93% in PDSA-1 (*n*=28; *p*=0.04). Then, there was a statistically significant decrease as compared to the baseline in PDSA-2 (*n*=6; mean 1.58%; *p*<0.001) and in PDSA-3 (*n*=21; mean 1.47%; *p*<0.0001). The primary outcome across the study period is illustrated in the SPC chart shown in Fig. 2. The baseline period and PDSA-1 met the criteria for special cause variation, i.e., a possible external influence of the system that would need investigation. An improvement was noted by PDSA-2 and 3, with no further special cause variation.

The themes that emerged from the baseline nursing staff qualitative survey (when HCPs were using baby monitors) were varied (Table 1). Positive findings included the facilitation of isolation door closure, increased HCP safety, and some positive effects on communication. Negative effects pertained to workflow and difficulty with the quality of communication, and they exceeded the positive findings in both frequency and intensity.

**Table 1:** Qualitative responses during baseline period

**Facilitation of isolation door closure**	
“Allowed room door to remain closed”	
**Increased healthcare worker safety**	
“Allows the team to minimize exposure to potentially infected COVID patients”	
**Positive effects on communication**	
“The clarity and quality of communication with the baby monitors is satisfactory most of the time”	
**Challenges with workflow**	
Proximity to device: “Person has to be close to the baby monitor in order to hear orders” Lack of portability: “Having to walk to the baby monitor to speak into it” Multiple people taking: “Radio communication ➔ information not being heard if both sides are talking the same time or one side starts talking and other side has not realized or heard”	
**Poor quality of communication**	
Ambient noise: “Hard to hear if lots of background noise or if department is busy” Poor clarity/static: “Hard to hear. Too much static” Hardware: “Requires occasionally troubleshooting, sound quality is often poor, especially through mask and shield. Issues with connecting between monitors”	

The qualitative themes identified post-PDSA-3 nursing staff survey included increased HCP safety, facilitation of door closure, positive communication effects, and increased patient confidentiality (Table 2). Moreover, positive effects were noted on workflow including being hands-free, improved efficiency, and portability. Themes also included some challenges with the new workflow, device design, and sound quality with the initial roll-out.

**Table 2:** Qualitative responses after PDSA-3

**Increased healthcare worker safety/stress**	
“Being able to have less people in the room during codes which makes it less stressful”	
**Facilitation of isolation door closure**	
“Good audio volume. Not needing to open main door. Having members on outside of code room being in communication loop”	
**Positive effects on workflow**	
Hands free: “Not having to push any buttons e.g. baby monitor to talk - constant communication (even when in medication room, etc.)” Efficiency in patient care: “Not having to leave the room. Orders received right away and initiated - no lag time” Portability: “I was still able to hear the communication inside of the room when I was away to get blood work supplies”	
**Challenges with workflow**	
Multiple/different groups of people speaking: “Sometimes people talk at the same time and it can get confusing at times” Difficulties with others not using transceiver: “Can't hear people not on transceiver --> maybe have one ear shorter?” Length of use: “Needs longer battery life for long codes”	
**Positive effects on communication**	
Clarity: “Clear communication between members wearing headsets. Range is very good (when in medication room the sound is clear)” Ambient Noise: “Can hear everyone clearly. No background noise.”	
**Increased patient confidentiality**	
“Definitely better than baby monitors in terms of patient confidentiality because people outside the room need not to shout for clearer communication”	
**Challenges with device design**	
“Uncomfortable after sometime”	
**Challenges with communication quality**	
“Sometimes the sound quality was not the best but improved with the new set”	

*PDSA* Plan-Do-Study-Act

**Fig. 3:**
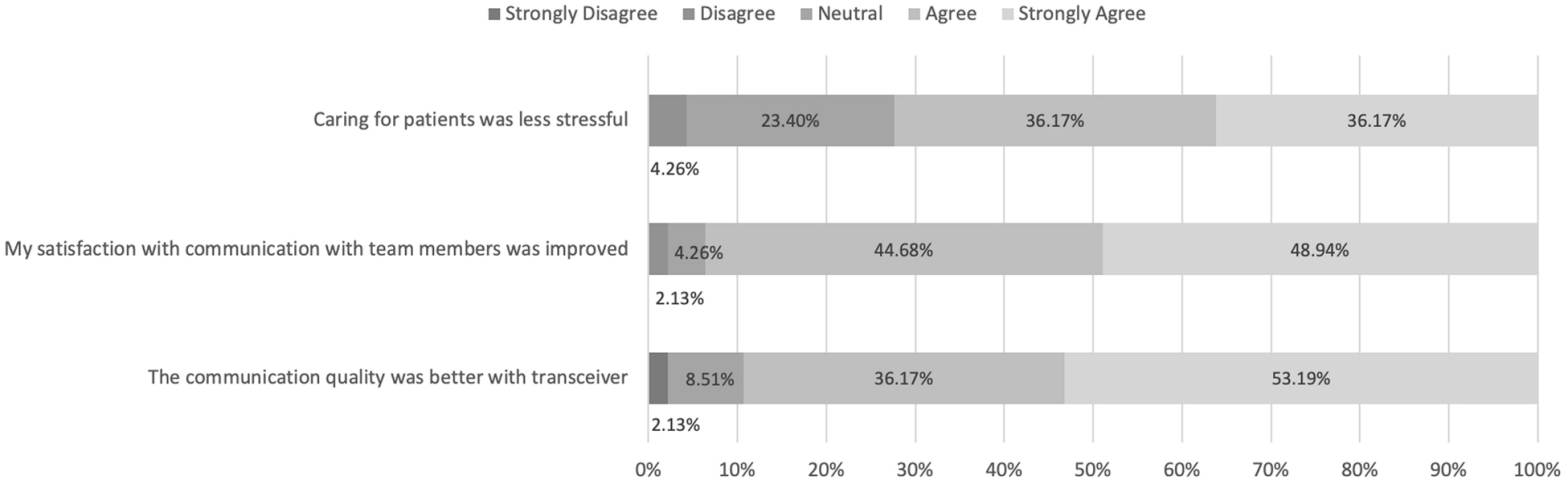
Post-PDSA-3 Likert survey questions. PDSA Plan-Do-Study-Act

Our secondary outcome of HCP-perceived sound clarity had a statistically significant improvement on the Likert survey (5 is best) from baseline (*n*=36; mean 3.36) to post-PDSA-3 (*n*=49; mean 4.21; *p*<0.001). Our three balancing measures of HCP-perceived errors, using other means of communication and alterations in workflow did not show any worsening; in fact, they all showed statistically significant improvements (Table 3). The post-PDSA-3 survey also showed the majority of respondents agreeing or strongly agreeing with utilizing the transceiver in making care for their patients less stressful, improved satisfaction with communication, and improved quality as compared to the previously used baby monitor (Fig. 3).

**Table 3:** Baseline and post-PDSA-3 nurse Likert survey

Measure	Survey item	Baseline mean (n=36)	Post-PDSA 3 mean (***n***=49)	***P*** value
*Outcome*	*Communication clarity*	*3.36*	*4.21*	*<0.001*
Balancing	Errors	2.61	2.13	0.026
Balancing	Door opening	3.00	2.06	<0.001
Balancing	Workarounds	3.89	2.46	<0.001
Balancing	Alerting workflow	4.08	2.21	<0.001

*PDSA* Plan-Do-Study-Act; Survey Items correspond to Appendix 2

Throughout the PDSA cycles, specific feedback was also obtained to improve the transceiver system and better integrate it into the workflow. Feedback and resultant actions are illustrated in Table 4. Finally, changes in patients presenting to the ED who failed the infectious screen are shown in Additional file 1: Appendix 3 and appear to show a slow general decline in patients meeting the criteria as the study period progressed.

**Table 4:** Feedback and actions for transceiver system improvement

Period	Feedback	Resultant actions
**Baseline period**	Bulky headsets; casing not robust	Changed casing–decreased size of the headset
	Foam ear covers (infection control)	Eliminated foam ear covers Staff educated on device cleaning
	Large ambient noise	Noise cancellation optimization
	Add an extra speaker in the room	Adding a speaker was not found to be effective
	Long wire connecting the headset	Removed wire and mounted device on the headset
**PDSA 1**	Battery life short for long resuscitations	Improved battery life to 2 h Staff educated on device docking and recharging
	Static noted	Adjusted settings to decrease static and ambient noise
	Improve storage and accessibility	New storage units placed beside accessible areas in the ED
**PDSA 2**	Not enough headsets per room for workflow	Increased the number of headsets per room to 6 headsets
	Casing handling issue	Casing upgraded for more robust materials
**PDSA 3**	Multiple people talking at once	Working towards possible 1 ear headset Working towards simulation exercises integrating headsets and new communication patterns

## Discussion

Our QI project with [1] HCP education, [2] the introduction of the transceiver, and [3] engagement through a local nurse champion substantially decreased door opening for infectious patients beyond the *apriori* 50% aim (primary outcome). Our secondary outcome also showed a significant improvement, but narrowly missing our a priori one Likert scale point. This improvement in communication was also supported by qualitative feedback, which demonstrated a positive experience with the transceiver as a communication device. While there have been many reports of frontline HCPs using baby monitors in the ED [23], operating room [24], and intensive care units [25], our qualitative surveys revealed substantial problems with their use pertaining to communication clarity and workflow, especially in high-acuity situations when they are even more important for safe patient care.

The special cause variation in the baseline period (Fig. 2) coincided with the addition of new curtains placed in front of isolation room glass doors, which was organized by ED leadership and was unrelated to the conduct of our project. As a result of this increased privacy, HCPs often kept the glass doors open, thereby posing a potential risk to others. A significant decrease in door opening was noted after the introduction of the transceiver by PDSA-2, and it was maintained through PDSA-3. A search for potential confounders leading to this notable decrease such as a new local policy, incentive, or critical incident did not reveal any contributors.

The educational intervention was not noted to have a difference on decreased door opening, but we posit it may have helped prime the participants towards further PDSA cycles along with the transceiver introduction. Moreover, we leveraged a local nurse champion in PDSA-3 to further support and coach HCPs in keeping with the technology acceptance model [26]. While this was anecdotally appreciated, a further change was not captured on the SPC chart given the already low values by PDSA-2.

We coupled the introduction of the transceiver with ongoing feedback. The introduction of (ICT) into a complex work ecology such as in the ED necessitates ongoing stakeholder engagement and feedback, also referred to as participatory design [27]. This feedback led to ongoing improvements in design and use as illustrated in Table 4. Moreover, key factors that enable the successful adoption and integration of ICTs in healthcare include portability, user satisfaction, and communicability [28]. The main focus of the QI approach was to engage relevant stakeholders at every stage of the project, thereby optimizing adoption and integration into the workflow. A large proportion of HCPs found that using the transceiver decreased their stress during patient care and increased their satisfaction in communication with team members.

Qualitative surveys also highlighted further improvement opportunities, such as optimizing communication strategies with multiple team members speaking in parallel. While allowing continuous communication for all users (instead of only one at a time like talkie-walkie push-to-talk systems) facilitated efficient communication, there was some confusion when multiple people spoke at the same time, as would be inherent in real-time in-person interactions. To optimize continuous communication and shared mental models for resuscitation patients [29], in situ simulation [30] may improve team performance.

### Limitations

Our pragmatic QI approach used a convenience sample based on the research coordinator availability. We also did not collect patient demographics (to reduce risk to participants), which may limit generalizability. Moreover, (CC) who developed the transceiver was not involved in study design, data collection, and data analysis to mitigate conflict of interest. Data collection was done as soon as patients entered the isolation rooms prior to the performance of infectious illness screening done by nurses, which led to a proportion of encounters being excluded. Moreover, we could not identify an adequate validated survey for our initiative, so a new instrument was developed to capture important measures instead.

The SPC chart shows special cause variation in the baseline period. Methodologically, the baseline should extend until no further special cause variation is noted. However, a change in practice was noted (the addition of curtains, leading to greater door opening), which highlighted the need to proceed for potential HCP safety. We elected to continue with PDSA-1 after seeing the following week of baseline data stabilize.

## Conclusion

Our iterative QI approach with HCP education, transceiver introduction, and leveraging a local champion demonstrated significant decreases in the door opening and improvement in communication clarity without increasing communication errors, workarounds, or alterations in the workflow. Future work may include scaling this initiative to other EDs, operating rooms, and intensive care units.

## Supplementary Information

**Additional file 1.** 

## Data Availability

The datasets during and/or analyzed during the current study are available from the corresponding author on reasonable request.

## Abbreviations

HCP: Healthcare provider
SARS-COV-2: Severe acute respiratory syndrome - coronavirus 2
ED: Emergency department
QI: Quality improvement
PDSA: Plan-Do-Study-Act
AGMPs: Aerosol-generating medical procedures
PPE: Protective equipment
MSH-UHN: Mount Sinai Hospital - University Health Network
ICT: Information communication technology
SPC: Statistical Process Control
