# Supplementary material for: Improving safety and communication for healthcare providers caring for SARS-COV-2 patients
Source: Int J Emerg Med. 2022 Nov 12;15:62. doi: 10.1186/s12245-022-00464-y (PMC9652974; doi:10.1186/s12245-022-00464-y)
Supplement: Supplementary file 1 — Additional file 1. [file 12245_2022_464_MOESM1_ESM.pdf]

Improving safety and communication for healthcare providers caring for SARS-COV-2 patients

Appendix

Appendix 1 – Triage Infectious Screening Questionnaire

1. Have you been asked to self isolate by Toronto Public Health due to exposure to a confirmed case of COVID-19?
2. Have you recently tested positive for COVID-19?
3. Have you had any of the following symptoms in the past 14 days?
    - Fever
    - Cough
    - Shortness of breath
    - Sore throat
    - Runny nose
    - Body aches
    - Eye pain
    - Headache
    - Severe fatigue
    - Vomiting
    - Diarrhea
    - Loss of smell of taste

Appendix 2. Baseline Survey Questions

**Q.1** Thinking of your recent experience in the ED (with the baby monitor system), how would you rate the following:<br><br>Strong disagree Disagree Neutral Agree Strongly Agree<br><br>*1* *2* *3* *4* *5*<br><br>1. Communication was clear between the teams inside and outside the closed doors.<br>2. There were errors in communication between the teams inside and outside the closed doors.<br>3. The isolation door had to be opened for communication.<br>4. The team had to use other means (shouting through closed door, hand gestures, or use a board for communication) between the teams inside and outside the closed doors.<br>5. I had to pause what I am doing clinically and get closer to the baby monitor to speak into it or listen.
**Q2.** Can you provide details of what worked well with your experience with the baby monitor system
**Q3.** Can you provide details of what didn't work well with your experience with the baby monitor system

Appendix 3 – Percentage of daily ED patients failing triage screen

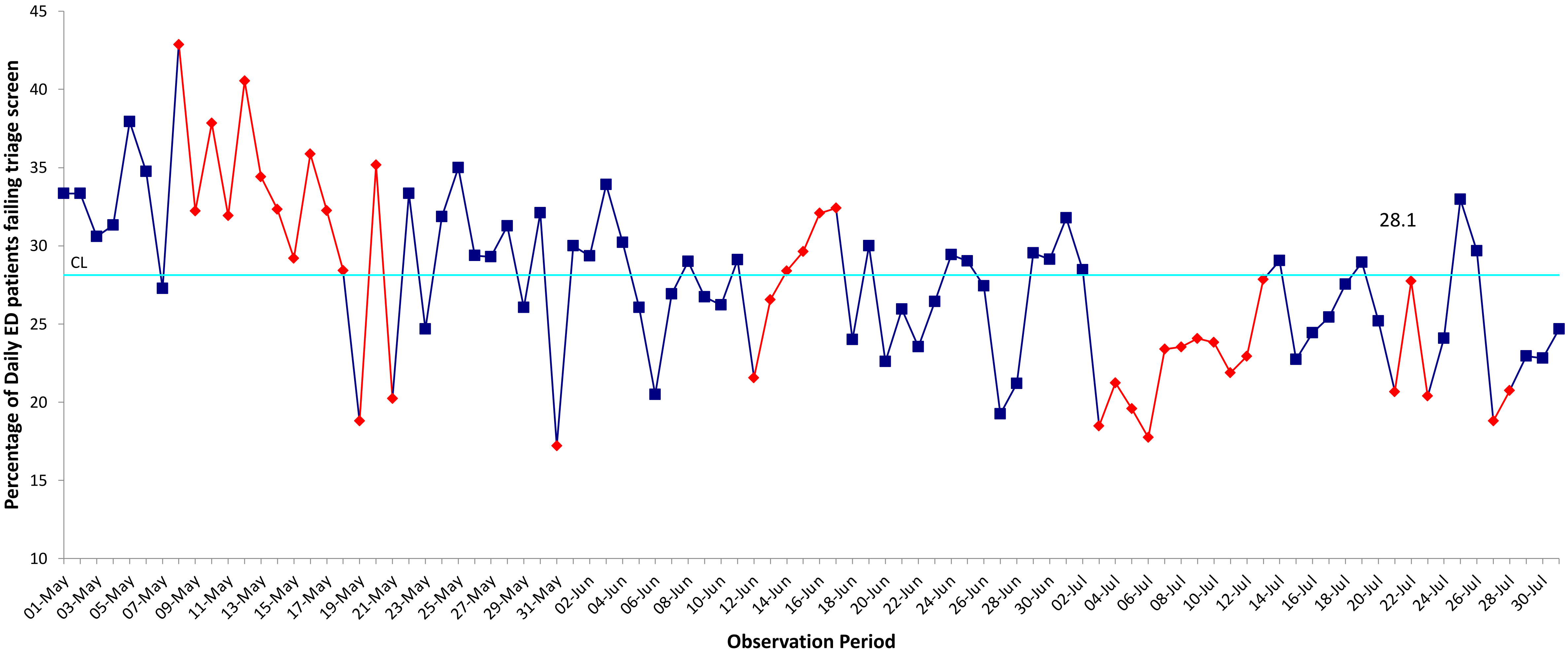

CL – Control Line (median)
